# Computationally driven discovery of phenyl(piperazin-1-yl)methanone derivatives as reversible monoacylglycerol lipase (MAGL) inhibitors

**DOI:** 10.1080/14756366.2019.1571271

**Published:** 2019-01-30

**Authors:** Giulio Poli, Margherita Lapillo, Vibhu Jha, Nayla Mouawad, Isabella Caligiuri, Marco Macchia, Filippo Minutolo, Flavio Rizzolio, Tiziano Tuccinardi, Carlotta Granchi

**Affiliations:** a Department of Pharmacy, University of Pisa, Pisa, Italy;; b Pathology Unit, Department of Molecular Biology and Translational Research, National Cancer Institute and Center for Molecular Biomedicine, Aviano, Italy;; c Department of Molecular Science and Nanosystems, Ca' Foscari Università di Venezia, Venezia, Italy;; d Sbarro Institute for Cancer Research and Molecular Medicine, Center for Biotechnology, College of Science and Technology, Temple University, Philadelphia, PA, USA

**Keywords:** MAGL inhibitors, virtual screening, molecular modelling, drug design

## Abstract

Monoacylglycerol lipase (MAGL) is an attractive therapeutic target for many pathologies, including neurodegenerative diseases, cancer as well as chronic pain and inflammatory pathologies. The identification of reversible MAGL inhibitors, devoid of the side effects associated to prolonged MAGL inactivation, is a hot topic in medicinal chemistry. In this study, a novel phenyl(piperazin-1-yl)methanone inhibitor of MAGL was identified through a virtual screening protocol based on a fingerprint-driven consensus docking (CD) approach. Molecular modeling and preliminary structure-based hit optimization studies allowed the discovery of derivative **4**, which showed an efficient reversible MAGL inhibition (IC_50_ = 6.1 µM) and a promising antiproliferative activity on breast and ovarian cancer cell lines (IC_50_ of 31–72 µM), thus representing a lead for the development of new and more potent reversible MAGL inhibitors. Moreover, the obtained results confirmed the reliability of the fingerprint-driven CD approach herein developed.

## Introduction

**Figure 1.**
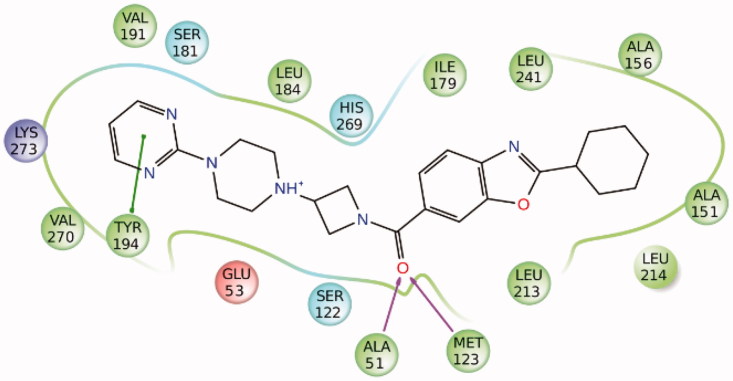
Schematic representation of the ZYH-MAGL interactions. Hydrophobic, polar, negative and positive charged residues are coloured green, sky blue, red, and violet, respectively.

The endocannabinoid system (ECS) is constituted by the cannabinoid receptors type 1 and type 2 (CB1, CB2), a series of signalling molecules called endocannabinoids (eCBs) and biosynthetic and degrading enzymes involved in the production and transformation of the eCBs. Anandamide (AEA) and 2-arachidonoylglycerol (2-AG) are the two main eCBs,^1^ which are synthesized on-demand in the plasma membrane and released into the extracellular space. After activating the cannabinoid receptors, eCBs are transported into the cytoplasm and degraded by specific enzymes. AEA is hydrolyzed by fatty acid amide hydrolase (FAAH) to arachidonic acid and ethanolamine, whereas 2-AG is predominantly hydrolyzed to arachidonic acid and glycerol by monoacylglycerol lipase (MAGL) and to a lesser extent by α/β hydrolase-6 and -12 (ABHD6 and ABHD12). The inhibition of eCBs degradation can be considered as a promising pharmacological strategy to activate the ECS limiting the side effects associated with direct receptor agonists.^2–4^ In addition, MAGL plays a key role in the progression and maintenance of cancer, being overexpressed in many aggressive tumour types. MAGL-catalyzed hydrolysis of monoacylglycerols in the peripheral tissues, such as adipose tissue and liver, provides a pool of free fatty acids, which constitute the building blocks for the formation of cellular membranes of growing tumour cells and for the synthesis of pro-tumorigenic signalling factors.^5^ Therefore, MAGL is an attractive therapeutic target for many pathologies such as neurodegenerative diseases, chronic pain, inflammatory pathologies as well as cancer. In the last years, many MAGL inhibitors were developed, of both synthetic and natural origin,^6^ ^,^ ^7^ although many of them were characterized by an irreversible mechanism of action that hindered their potential subsequent clinical development. In fact, a prolonged MAGL inactivation provokes a chronic increase of 2-AG, which is the endogenous agonist of cannabinoid receptor CB1. The continuous CB1 stimulation by elevated 2-AG concentrations has negative effects, such as loss of therapeutic effects and physical dependence.^8^ Reversible MAGL inhibitors are devoid of these problems and therefore they represent a safer alternative to irreversible inhibitors.^9–11^ Recently, we tested the reliability of the consensus docking (CD) protocol combining different docking methods. Our results showed that this approach was able to predict ligand binding poses better than the single docking procedures and showed to be a promising strategy for improving performance and hit rates of virtual screening (VS) campaigns.^12^ ^,^ ^13^ This CD protocol has already been successfully employed for the identification of new non-covalent fatty acid amide hydrolase (FAAH) inhibitors^14^ and novel salicylate synthase (MbtI) furanic inhibitors.^15^ However, due to a sort of symmetry in MAGL binding site, we have recently verified that docking procedures for MAGL inhibitors are not always able to identify a unique ligand-binding disposition, suggesting the presence of two possible orientations that are geometrically opposite but nevertheless equivalent in terms of ligand–protein interactions.^9^ As shown in Figure 1, illustrating the interactions of (2-cyclohexyl-1,3-benzoxazol-6-yl){3-[4-(pyrimidin-2-yl)piperazin-1-yl]azetidin-1-yl}methanone (ZYH) with MAGL (3PE6 PDB code),^16^ the enzyme binding site can be roughly schematized as an ellipsoidal shaped cavity where the central residues A51 and M123 form two H-bonds with the carbonyl group of the ligand and all surrounding residues show lipophilic interactions with the inhibitor. In particular, the 2-cyclohexylbenzo[*d*]oxazole fragment of the molecule is inserted into a lipophilic pocket mainly delimited by A151, A156, I179, L213, L214, and L241, whereas the pyrimidine ring establishes a π-π stacking with Y194 and other hydrophobic interactions with L184, V191, and V270. On the basis of the symmetrical features of MAGL binding site, the CD approach might lead to a high number of false negatives, since only compounds showing a unique preferred binding orientation are proposed as potential active ligands. On the other hand, we recently demonstrated that receptor-based fingerprint analysis can improve docking reliability.^17^ For this reason, we decided to develop a VS protocol based on a fingerprint-driven CD approach, in order to identify novel compounds endowed with MAGL inhibitory activity.

## Materials and methods

### FLAP ligand-based preliminary filter

The Chembridge database (XPRESS-Pick™ stock database, about 480 000 compounds) was used as the compounds database. The screening was performed by using ZYH as a template structure. Calculations were run by leaving all settings as their defaults, i.e. (a) using only one conformer of the query structure as a template, (b) considering only the fingerprint matching and (c) using a low level of accuracy.

### Fingerprint-based CD analysis

Four different docking procedures were applied and for each docking calculation only the best-scored pose was taken into account. The docking calculations were carried out by using Autodock 4.2.3,^18^ Dock 6.7,^19^ Fred 3.0,^20^ and GOLD 5.1 (with ChemPLP fitness function),^21^ employing the procedures previously described.^22^ ^,^ ^23^ The ligands were docked into the binding site of human MAGL (3PE6 PDB code^16^) by using the different docking procedures. For each docking pose the interaction fingerprint was evaluated using Binana software,^24^ summarizing the main interactions between the ligand and MAGL binding site. Then, by using an in-house software, only compounds that in the binding poses predicted by all four docking procedures showed the desired interaction fingerprints (i.e the π–π stacking interaction with Y194, the lipophilic interactions with L213 and L241 and at least one H-bond with A51 and M123) were selected and subjected to molecular dynamic (MD) simulations.

### MD simulations

All simulations were performed using AMBER, version 16.^25^ MD simulations were carried out using the ff14SB force field at 300 K in a rectangular parallelepiped water box, following a protocol already used for pose prediction studies of MAGL inhibitors.^26^ The TIP3P explicit solvent model for water was used. Sodium ions were added as counter ions to neutralize the system. Prior to MD simulations, the complexes were energy minimized through 5000 steps of steepest descent followed by conjugated gradient. Particle mesh Ewald electrostatics and periodic boundary conditions were used in the simulation. The time step of the simulations was 2.0 fs with a cutoff of 10 Å for the nonbonded interactions, while SHAKE was employed to keep all bonds involving hydrogen atoms rigid. Constant-volume periodic boundary MD was carried out for 0.5 ns, during which the temperature was raised from 0 to 300 K. The system was then equilibrated through 3 ns of constant pressure periodic boundary MD, carried out at 300 K using the Langevin thermostat to maintain constant the temperature of our system. In these two MD steps, all the α carbons of the protein were blocked with a harmonic force constant of 10 kcal/mol Å^2^. Eventually, 26.5 ns of constant pressure MD simulation were performed without any position restraint. General Amber force field (GAFF) parameters were assigned to the ligands, while partial charges were calculated using the AM1-BCC method as implemented in the Antechamber suite of AMBER 16.

### Binding energy evaluation

The evaluation of the binding energy associated to the ligand–protein complexes analyzed through MD simulations was carried out using AMBER 16 as already reported.^27–29^ The trajectories relative to the last 20 ns of each simulation were extracted and used for the calculation, for a total of 200 snapshots (at time intervals of 100 ps). Van der Waals, electrostatic, and internal interactions were calculated with the SANDER module of AMBER 16, whereas polar energies were calculated using the Poisson − Boltzmann method with the MM-PBSA module of AMBER 16. Dielectric constants of 1 and 80 were used to represent the gas and water phases, respectively, while the MOLSURF program was employed to estimate the nonpolar energies.

### MAGL inhibition assays

Compounds **1–3** were purchased from ChemBridge corporation, whereas compounds **4** and **5** were synthesized. Human recombinant MAGL, 4-nitrophenyl acetate substrate (4-NPA) and **CAY10499** were purchased from Cayman Chemical. The IC_50_ values were generated in 96-well microtiter plates. The MAGL reaction was conducted at rt at a final volume of 200 µL in 10 µM Tris buffer, pH 7.2, containing 1 µM EDTA. A total of 150 µL of 4-NPA 133.3 µM was added to 10 µL of DMSO containing the appropriate amount of compound. The reaction was initiated by the addition of 40 µL of MAGL (11 ng/well) in such a way that the assay was linear over 30 min. The final concentration of the analyzed compounds ranged from 10 to 0.00001 µM for **CAY10499** and from 200 to 0.0128 µM for the other compounds. After the reaction had proceeded for 30 min, absorbance values were then measured by using a VictorX3 PerkinElmer instrument at 405 nm. Two reactions were also run: one reaction containing no compounds and the second one containing neither inhibitor nor enzyme. To remove possible false positive results, a blank analysis was carried out for each compound concentration and the final absorbance results were obtained detracting the absorbance produced by the presence of all the components except MAGL in the same conditions.

### Cell viability assay

Human breast MDA-MB-231, colorectal HCT116 and ovarian CAOV3, OVCAR3, and SKOV3 cancer cells (from ATCC) were maintained at 37 °C in a humidified atmosphere containing 5% CO_2_ according to the supplier. Cells (5 × 10^2^) were plated in 96-well culture plates. The day after seeding, vehicle or compounds were added at different concentrations to the medium. Compounds were added to the cell culture at a concentration ranging from 200 to 0.02 μM. Cell viability was measured after 96 h according to the supplier (Promega, cat. n° G7571) with a Tecan M1000 PRO instrument. IC_50_ values were calculated from logistical dose-response curves. Averages were obtained from triplicates and error bars are standard deviations.

## Results and discussion

As already reported, the main Achilles’ heel of the CD approach is the computing time required. In fact, by using this method a whole data set of molecules should be subjected to multiple docking procedures. For this reason, large libraries of compounds require large amount of CPU time. One of the possible solutions for this problem could be the application of a pre-filtering step able to decrease the number of compounds to be analyzed. Recently, we verified the reliability of FLAP software^30^ in VS studies;^14^ ^,^ ^31^ therefore, we used this software as a pre-filter for selecting potential reversible MAGL inhibitors. The Chembridge database (XPRESS-Pick™ stock database) was subjected to a ligand-based bit-string filtering using as a template the crystal structure of ZYH extracted from the complex with MAGL (3PE6 PDB code).^16^ In a ligand-based bit-string analysis, FLAP compares the template molecule with the whole data set of compounds through the comparison of the molecular interaction fields (MIFs)-based pharmacophoric features translated into fingerprints. The test compounds are then scored on the basis of their fingerprint similarity with respect to the template molecule. By applying this procedure and considering compounds with a Glob-sum score (representing the overall fingerprint similarity) higher than 1.5, 13772 compounds were selected as potential MAGL inhibitors and thus subjected to the CD studies. The docking step was carried out by employing Gold (ChemPLP fitness function), Dock, Fred, and Autodock software, as they already showed reliable results for identifying new MAGL inhibitors.^22^ In a classical CD procedure, the four docking poses obtained for each compound (resulting from the calculations carried out by using the four docking procedures) should be clustered together to search for common binding modes. However, as mentioned in the introduction, MAGL binding site shows a sort of symmetry and, therefore, compounds can interact with the enzyme by assuming two opposite, but yet equivalent, dispositions making the CD approach not particularly reliable. Therefore, instead of applying the classical CD protocol, a fingerprint-based CD approach was tested. More in detail, the interaction of ZYH with MAGL was analyzed and converted into a fingerprint code by means of Binana software.^24^ The main ligand–protein interactions identified, i.e. the two H-bonds with A51 or M123, the π–π stacking with Y194 and the lipophilic interactions with L213 and L241, were then considered as fundamental for the inhibitory activity (see Figure 1). Following this hypothesis, all the docking poses previously generated were processed by using Binana and the resulting fingerprints were analyzed employing an in-house software. Only the compounds that showed the π–π stacking interaction with Y194, the lipophilic interactions with L213 and L241 and at least one H-bond with A51 and M123 in the binding poses predicted by all four docking procedures, were then considered as potential active MAGL inhibitors. By applying this kind of approach, the compounds that showed different binding dispositions were still considered as potential active compounds, provided that the above-described interactions were displayed by all the different dispositions. Therefore, this kind of optimization of the CD approach should be able to solve the problem generated by the presence of symmetrical binding sites. Following this fingerprint-based CD step only 17 compounds were selected, as they were the only compounds that showed the desired interactions with MAGL in the binding poses predicted by all four docking methods. The low number of compounds that survived this selection step was in agreement with previous studies reported in the literature, where CD was combined with pharmacophore-based/receptor-based post-docking filters, which highlighted the high strictness of this type of approach.^15^ ^,^ ^32^ The 17 compounds obtained by the previous VS steps were subjected to a total of 30 ns of MD simulation using the Gold docking pose as the starting ligand orientation and for compounds showing different binding dispositions all of them were studied. The MD trajectories of these ligand–protein complexes were thus analyzed in terms of H-bond stability and Root-Mean Squared Deviation (RMSD) of the ligand disposition. Only the three compounds maintaining at least one of the two H-bonds with A51 and M123 for 80% of the simulation and showing an average RMSD of their binding pose < 2.0 Å were selected and further considered.

These three compounds were thus purchased and subjected to a MAGL inhibition assay together with the reference MAGL covalent inhibitor **CAY1049910**, which was used as a positive control.^33^ As indicated in Table 1, compound **1** showed an appreciable MAGL inhibitory activity, with an IC_50_ value of 23.3 µM, whereas compounds **2** and **3** were not able to inhibit MAGL at concentrations lower than 100 µM.

**Table 1. t0001:** Structure and MAGL inhibition activity of the tested compounds.

		

**Figure 2.**
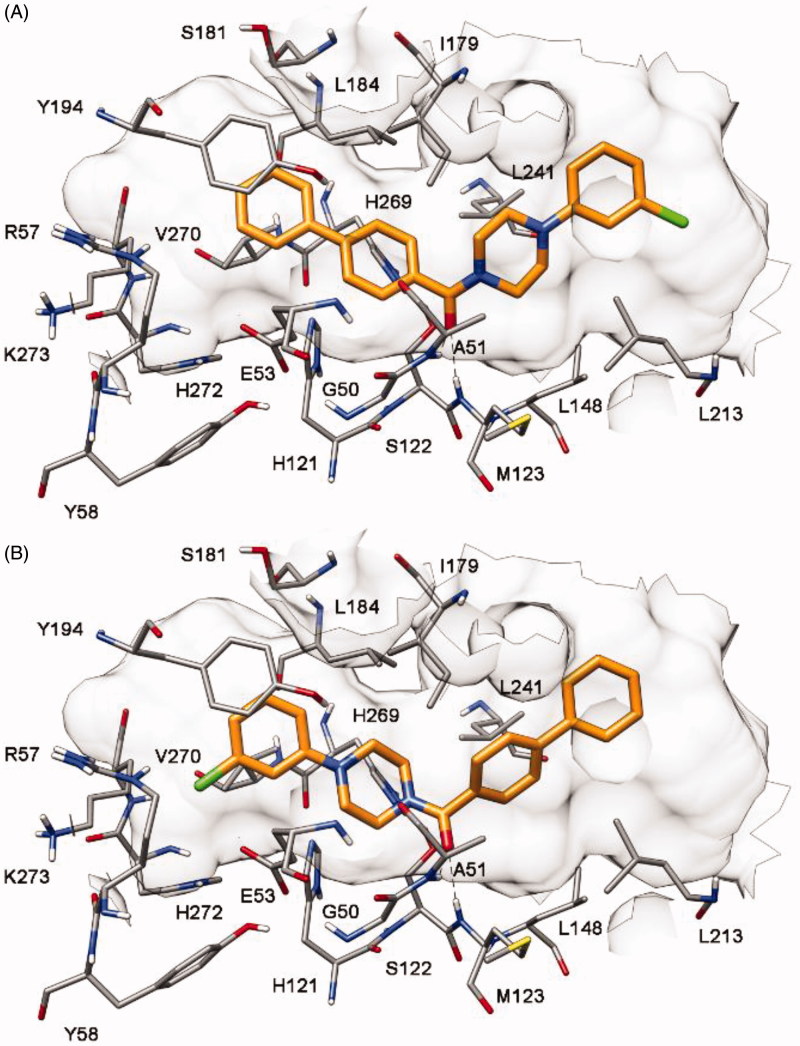
Docking of compound **1** into MAGL. (A) Binding mode A; (B) binding mode B.

The docking analysis for compound **1** highlighted the presence of two possible binding dispositions of the ligand. As shown in Figure 2, in both binding orientations (A and B) the ligand formed two H-bonds with the backbone nitrogen of A51 and M123 through its carbonyl oxygen, while a π-π stacking with Y194 and lipophilic interactions with both L213 and L241 were established by the terminal phenyl rings of the molecule. In order to assess the reliability of the two possible binding modes from an energetic point of view, ligand–protein binding energy evaluations were performed on the MD trajectories relative to the last 20 ns of simulation by using the Molecular Mechanic-Poisson Boltzmann surface area (MM-PBSA) methods. Based on this evaluation (see [Table t0001] Supplementary Table S1 ), binding mode A appeared to be the most reliable, since its estimated binding energy (ΔPBSA = −30.1 kcal/mol) was found to be higher than that associated to binding mode B (ΔPBSA = −28.6 kcal/mol). However, the energy difference was rather modest and could not allow a reliable identification of the most probable binding orientation for this compound.

**Figure 3.**
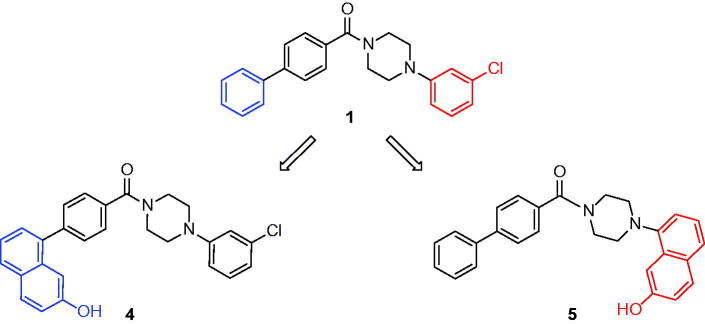
Synthesized compounds **4** and **5**. In blue or in red are highlighted the portions of compound **1** that are substituted by a 7-hydroxynaphthalen-1-yl moiety in compounds **4** and **5**.

Therefore, to experimentally provide additional data for identifying the preferred binding disposition of compound **1**, we planned to synthesize two derivatives that we assumed could be able to respectively adopt only one of the two binding modes proposed by the molecular modelling analysis (see Supplementary Material for details). In fact, compounds **4** and **5** (Table 1) were designed by considering the possible H-bond network that they could form with residues E53 and H272. In particular, these two compounds were characterized by the presence of a 7-hydroxynaphthalen-1-yl fragment that replaces the unsubstituted phenyl (**4**, Figure 3) and the 3-chlorophenyl (**5**, Figure 3) ring of compound **1** and this moiety should be able to interact with residues E53 and H272.

**Figure 4.**
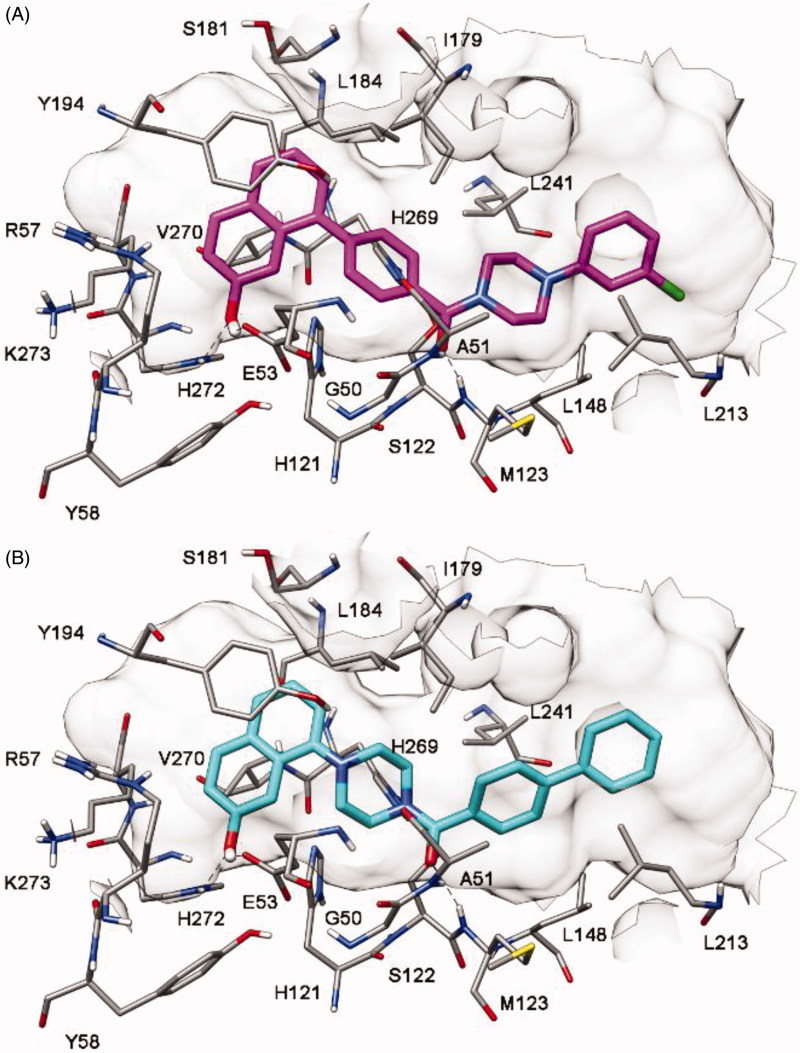
Docking results of compounds **4** (A) and **5** (B) into MAGL.

As shown in Figure 4, our hypothesis was supported by docking studies, as only a single binding disposition was predicted for each compound, with the 7-hydroxynaphthalen-1-yl fragment forming H-bonds with E53 and H272. Because of this disposition, compound **4** showed binding mode A whereas compound **5** adopted binding mode B of Figure 4.

The two compounds were thus synthesized (Supplementary Material) and tested for their MAGL inhibition activity. As shown in Table 1, in agreement with the docking results, both binding modes proved to be possible because both compounds showed inhibition of MAGL activity. However, binding mode A was confirmed to be the preferred one, because compound **4** demonstrated a 5-fold higher activity (IC_50_ = 6.1 μM) than compound **5** (IC_50_ = 31.9 μM). Furthermore, the 4-fold improvement of activity obtained with compound **4**, with respect to the starting compound **1**, supported the binding disposition suggested for this class of compounds. As shown in Figure 4(A), the compound maintains the interactions required by the fingerprint-based filter, forming H-bonds with both A51 and M123, a π-π stacking with Y194 and lipophilic interactions with L213 and L241 (as well as with L148, L184, I179, and V270). Moreover, the 7-hydroxy group of the ligand shows two additional H-bonds with E53 and H272 that support its higher activity with respect to compound **1**.

**Figure 5.**
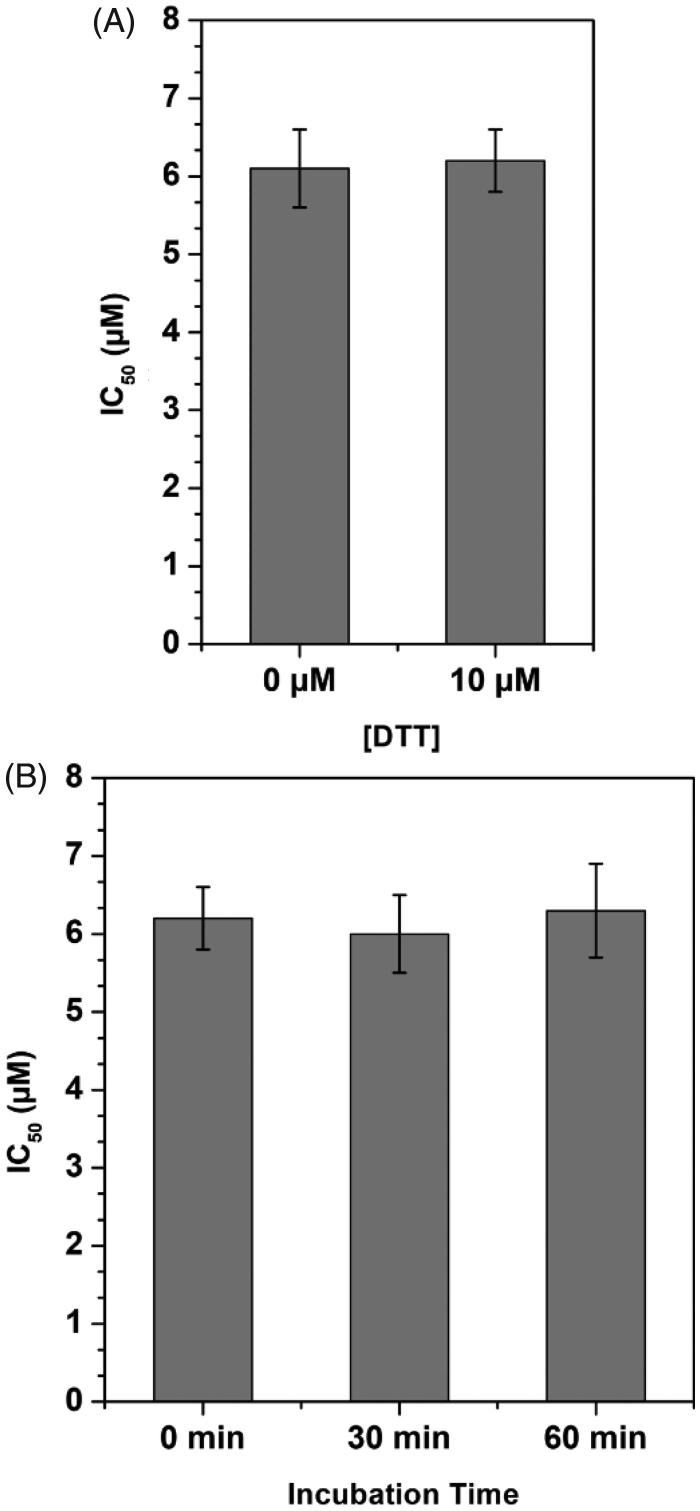
Compound **4**-MAGL inhibition analysis. A) Effect of DTT on MAGL inhibition activity. B) IC_50_ (µM) values of **4** at different preincubation times with MAGL (0 min, 30 min and 60 min).

In order to verify whether the compounds could interact with cysteine residues of MAGL enzyme, the activity of the most potent inhibitor **4** was also tested in presence of the thiol-containing agent 1,4-dithio-dl-threitol (DTT). As shown in Figure 5(A), the IC_50_ value of compound **4** was only very slightly, but not significantly, influenced by the presence of DTT, shifting from 6.1 μM when assayed in the absence of DTT to 6.2 μM when assayed in the presence of 10 μM DTT, thus excluding any significant interaction with MAGL cysteine residues. Furthermore, with the aim of establishing whether the mechanism of inhibition was reversible or irreversible, the effects of preincubation on the inhibitory activity of compound **4** were evaluated. In this assay, the compound was preincubated with the enzyme for 0, 30, and 60 min before adding the substrate to start the enzymatic reaction. An irreversible inhibitor should show a higher potency after longer incubation times, whereas a reversible inhibitor should display a constant inhibition potency that is independent from the incubation time. As shown in Figure 5(B), this assay confirmed the reversible property of **4**, as it did not show any significant increase in inhibitory potency at longer incubation times.

Finally, compound **4** was also selected for further *in vitro* experiments to evaluate its anticancer potency against cancer cells. The reference compound **CAY10499** was also included in the experiments. Due to the key role that MAGL plays in the progression of breast, colon, and ovarian cancer, five tumour cell lines were chosen: human breast MDA-MB-231, colorectal HCT116 and ovarian CAOV3, OVCAR3, and SKOV3 cancer cells (Table 2).^34^ Derivative **4** produced an appreciable inhibition of cell viability in all the tested cell lines, with IC_50_ values ranging from 31 to 72 µM. With respect to the covalent reference inhibitor **CAY10499**, compound **4** showed a very similar antiproliferative efficacy in HCT116 and SKOV3 cancer cells, and it was even slightly more potent in MDA-MB-231 and CAOV3 cells, with a lower potency only for what concerns the OVCAR3 cell line. These results suggest that the phenyl(piperazin-1-yl)methanone could be an interesting scaffold to be further explored for the identification of novel reversible MAGL inhibitors.

**Table 2.** Cell viability inhibitory activities (IC_50_ values) of compounds **4** and **CAY10499**.

IC_50_ (µM, mean ± SD)					
Compound	HCT116	MDA-MB-231	CAOV3	OVCAR3	SKOV3
4	48 ± 2	59 ± 5	51 ± 3	72 ± 4	31 ± 2
CAY10499	45 ± 3	82 ± 5	90 ± 6	52 ± 3	38 ± 4

In conclusion, we herein reported a VS study relying on a fingerprint-based CD approach focused on the identification of novel reversible MAGL inhibitors. This first step of the study led to the discovery of compound **1** as an interesting MAGL inhibitor. Then, molecular modelling studies guided chemical modifications of the structure of the initial hit compound **1** in order to establish the binding orientation of this ligand. This preliminary analysis highlighted the most probable binding orientation of this class of compounds and led to the discovery of compound **4** as a novel reversible MAGL inhibitor endowed with promising anticancer activity in breast and ovarian cancer cell lines, which can be considered as a lead for the development of new and more potent reversible MAGL inhibitors. Furthermore, these successful screening results suggest that the use of ligand–protein interaction fingerprints as a post-docking filter can compensate for the limitations encountered when applying the CD approach on protein targets characterized by a considerable level of symmetry within their binding site. The fingerprint-based CD protocol herein reported may be thus applied in future receptor-based VS studies aimed at developing small-molecule inhibitors of other therapeutically interesting targets.

## Supplementary Material

Supplemental Material

## References

[1] Macedonio G, Stefanucci A, Maccallini C, et al. Hemopressin peptides as modulators of the endocannabinoid system and their potential applications as therapeutic tools. Protein Pept Lett 2016;23:1045–51. 27748182 10.2174/0929866523666161007152435

[2] Ahn K, McKinney MK, Cravatt BF Enzymatic pathways that regulate endocannabinoid signaling in the nervous system. Chem Rev 2008;108:1687–707. 18429637 10.1021/cr0782067 PMC3150828

[3] Aghazadeh Tabrizi M, Baraldi PG, Ruggiero E, et al. Synthesis and structure activity relationship investigation of triazolo[1,5-a]pyrimidines as CB2 cannabinoid receptor inverse agonists. Eur J Med Chem 2016;113:11–27. 26922225 10.1016/j.ejmech.2016.02.032

[4] Stefanucci A, Macedonio G, Dvorácskó S, et al. Novel Fubinaca/Rimonabant hybrids as endocannabinoid system modulators. Amino Acids 2018;50:1595–605. 30145711 10.1007/s00726-018-2636-1

[5] Nomura DK, Long JZ, Niessen S, et al. Monoacylglycerol lipase regulates a fatty acid network that promotes cancer pathogenesis. Cell 2010;140:49–61. 20079333 10.1016/j.cell.2009.11.027 PMC2885975

[6] Scalvini L, Piomelli D, Mor M Monoglyceride lipase: structure and inhibitors. Chem Phys Lipids 2016;197:13–24. 26216043 10.1016/j.chemphyslip.2015.07.011 PMC4728057

[7] Granchi C, Caligiuri I, Minutolo F, et al. A patent review of monoacylglycerol lipase (MAGL) inhibitors (2013-2017). Expert Opin Ther Pat 2017;27:1341–51. 29053063 10.1080/13543776.2018.1389899

[8] Schlosburg JE, Blankman JL, Long JZ, et al. Chronic monoacylglycerol lipase blockade causes functional antagonism of the endocannabinoid system. Nat Neurosci 2010;13:1113–9. 20729846 10.1038/nn.2616 PMC2928870

[9] Granchi C, Rizzolio F, Palazzolo S, et al. Structural optimization of 4-chlorobenzoylpiperidine derivatives for the development of potent, reversible, and selective monoacylglycerol lipase (MAGL) inhibitors. J Med Chem 2016;59:10299–314. 27809504 10.1021/acs.jmedchem.6b01459

[10] Granchi C, Caligiuri I, Bertelli E, et al. Development of terphenyl-2-methyloxazol-5(4*H*)-one derivatives as selective reversible MAGL inhibitors. J Enzyme Inhib Med Chem 2017;32:1240–52. 28936880 10.1080/14756366.2017.1375484 PMC6009861

[11] Granchi C, Rizzolio F, Bordoni V, et al. Tuccinardi T. 4-Aryliden-2-methyloxazol-5(4*H*)-one as a new scaffold for selective reversible MAGL inhibitors. J Enzyme Inhib Med Chem 2016;31:137–46. 25669350 10.3109/14756366.2015.1010530

[12] Tuccinardi T, Poli G, Romboli V, et al. Extensive consensus docking evaluation for ligand pose prediction and virtual screening studies. J Chem Inf Model 2014;54:2980–6. 25211541 10.1021/ci500424n

[13] Poli G, Martinelli A, Tuccinardi T Reliability analysis and optimization of the consensus docking approach for the development of virtual screening studies. J Enzyme Inhib Med Chem 2016;31:167–73. 10.1080/14756366.2016.1193736 27311630

[14] Poli G, Giuntini N, Martinelli A, Tuccinardi T Application of a FLAP-consensus docking mixed strategy for the identification of new fatty acid amide hydrolase inhibitors. J Chem Inf Model 2015;55:667–75. 25746133 10.1021/ci5006806

[15] Chiarelli LR, Mori M, Barlocco D, et al. Discovery and development of novel salicylate synthase (MbtI) furanic inhibitors as antitubercular agents. Eur J Med Chem 2018;155:754–63. 29940465 10.1016/j.ejmech.2018.06.033

[16] Schalk-Hihi C, Schubert C, Alexander R, et al. Crystal structure of a soluble form of human monoglyceride lipase in complex with an inhibitor at 1.35 Å resolution. Protein Sci 2011;20:670–83. 21308848 10.1002/pro.596 PMC3081545

[17] Poli G, Jha V, Martinelli A, et al. Development of a fingerprint-based scoring function for the prediction of the binding mode of carbonic anhydrase II inhibitors. Int J Mol Sci 2018;19:1851. 10.3390/ijms19071851 PMC6073570 29937490

[18] Morris GM, Ruth H, Lindstrom W, et al. AutoDock4 and AutoDockTools4: automated docking with selective receptor flexibility. J Comput Chem 2009;30:2785–91. 19399780 10.1002/jcc.21256 PMC2760638

[19] Allen WJ, Balius TE, Mukherjee S, et al. 6: impact of new features and current docking performance. J Comput Chem 2015;36:1132–56. 25914306 10.1002/jcc.23905 PMC4469538

[20] McGann M FRED pose prediction and virtual screening accuracy. J Chem Inf Model 2011;51:578–96. 21323318 10.1021/ci100436p

[21] Verdonk ML, Cole JC, Hartshorn MJ, et al. Improved protein-ligand docking using GOLD. Proteins Struct Funct Genet 2003;52:609–23. 12910460 10.1002/prot.10465

[22] Tuccinardi T, Granchi C, Rizzolio F, et al. Identification and characterization of a new reversible MAGL inhibitor. Bioorganic Med Chem 2014;22:3285–91. 10.1016/j.bmc.2014.04.057 24853323

[23] Tuccinardi T, Poli G, Dell'Agnello M, et al. Receptor-based virtual screening evaluation for the identification of estrogen receptor β ligands. J Enzyme Inhib Med Chem 2015;30:662–70. 25265323 10.3109/14756366.2014.959946

[24] Durrant JD, McCammon JA BINANA: a novel algorithm for ligand-binding characterization. J Mol Graph Model 2011;29:888–93. 21310640 10.1016/j.jmgm.2011.01.004 PMC3099006

[25] Case DA, Babin V, Berryman JT, et al. AMBER, version 14. San Francisco (CA): University of California; 2015 Available from: http://ambermd.org/.

[26] De Leo M, Huallpa CG, Alvarado B, et al. New diterpenes from Salvia pseudorosmarinus and their activity as inhibitors of monoacylglycerol lipase (MAGL). Fitoterapia 2018;130:251–8. 30240845 10.1016/j.fitote.2018.09.010

[27] Dömötör O, Tuccinardi T, Karcz D, et al. Interaction of anticancer reduced Schiff base coumarin derivatives with human serum albumin investigated by fluorescence quenching and molecular modeling. Bioorg Chem 2014;52:16–23. 24291035 10.1016/j.bioorg.2013.10.003

[28] Milella L, Milazzo S, De Leo M, et al. α-Glucosidase and α-amylase inhibitors from arcytophyllum thymifolium. J Nat Prod 2016;79:2104–12. 27509358 10.1021/acs.jnatprod.6b00484

[29] Poli G, Lapillo M, Granchi C, et al. Binding investigation and preliminary optimisation of the 3-amino-1,2,4-triazin-5(2*H*)-one core for the development of new Fyn inhibitors. J Enzyme Inhib Med Chem 2018;33:956–61. 29747534 10.1080/14756366.2018.1469017 PMC6009924

[30] Baroni M, Cruciani G, Sciabola S, et al. A common reference framework for analyzing/comparing proteins and ligands. fingerprints for ligands and proteins (FLAP): theory and application. J Chem Inf Model 2007;47:279–94. 17381166 10.1021/ci600253e

[31] Poli G, Tuccinardi T, Rizzolio F, et al. Identification of new fyn kinase inhibitors using a FLAP-based approach. J Chem Inf Model 2013;53:2538–47. 24001328 10.1021/ci4002553

[32] Poli G, Scarpino A, Aissaoui M, et al. Identification of lactate dehydrogenase 5 inhibitors using pharmacophore-driven consensus docking. Curr Bioact Compd 2018;14:197–204.

[33] Muccioli GG, Labar G, Lambert DM CAY10499, a novel monoglyceride lipase inhibitor evidenced by an expeditious MGL assay. Chembiochem 2008;9:2704–10. 18855964 10.1002/cbic.200800428

[34] Bononi G, Granchi C, Lapillo M, et al. Discovery of long-chain salicylketoxime derivatives as monoacylglycerol lipase (MAGL) inhibitors. Eur J Med Chem 2018;157:817–36. 30144699 10.1016/j.ejmech.2018.08.038

