# Supplementary material for: Computationally driven discovery of phenyl(piperazin-1-yl)methanone derivatives as reversible monoacylglycerol lipase (MAGL) inhibitors
Source: J Enzyme Inhib Med Chem. 2019 Jan 30;34(1):589–96. doi: 10.1080/14756366.2019.1571271 (PMC6352951; doi:10.1080/14756366.2019.1571271)
Supplement: Supplemental Material [file IENZ_A_1571271_SM0805.pdf]

Computationally Driven Discovery of phenyl(piperazin-1-yl)methanone derivatives as reversible MAGL inhibitors.

Giulio Poli,[1] Margherita Lapillo,[1] Vibhu Jha,[1] Nayla Mouawad,[1,2] Isabella Caligiuri,[2] Marco Macchia,[1] Filippo Minutolo,[1] Flavio Rizzolio,[2,3] Tiziano Tuccinardi[1] Carlotta Granchi,[1,4]

[1]Department of Pharmacy, University of Pisa, Pisa, Italy, [2]Pathology Unit, Department of Molecular Biology and Translational Research, National Cancer Institute and Center for Molecular Biomedicine, Aviano (PN), Italy, [3]Department of Molecular Science and Nanosystems, Ca' Foscari Università di Venezia, Venezia-Mestre, Italy, [4]Sbarro Institute for Cancer Research and Molecular Medicine, Center for Biotechnology, College of Science and Technology, Temple University, Philadelphia, PA, USA

Address for correspondence: Tiziano Tuccinardi, Department of Pharmacy, University of Pisa, Via Bonanno 6, 56126 Pisa, Italy. E-mail: *tiziano.tuccinardi@unipi.it*

Table of Content

Chemistry Pags 2-9

MM-PBSA evaluation Pags 9

CHEMISTRY

General Procedures and Materials. All solvents and chemicals were used as purchased without further purification from Aldrich-Merck or Alfa Aesar-Thermo Fisher. Intermediate **11** was synthesized as previously reported.[1] Chromatographic separations were performed on silica gel columns by flash chromatography (Kieselgel 40, 0.040−0.063 mm; Merck). Reactions were followed by thin layer chromatography (TLC) on aluminum silica gel (60 F254) sheets that were visualized under a UV lamp. Evaporation was performed in vacuo (rotating evaporator). Sodium sulphate was always used as the drying agent. Proton (^{1}H) and carbon (^{13}C) NMR spectra were obtained with a Bruker Avance III 400 MHz spectrometer using the indicated deuterated solvents. Chemical shifts are given in parts per million (ppm) (δ relative to residual solvent peak for ^{1}H and ^{13}C). Yields refer to isolated and purified products derived from non-optimized procedures. HPLC analysis: compounds are ≥ 95% pure by HPLC, confirmed via UV detection (λ = 254 nm). Analytical reversed-phase HPLC was conducted using a Kinetex EVO C18 column (5 µm, 150 mm × 4.6 mm, Phenomenex, Inc.); eluent A, water; eluent B, CH_3CN; after 5 min at 25% B, a gradient was formed from 25% to 75% of B in 5 min and held at 75% of B for 10 min; flow rate was 1 mL/min.

Synthesis of compound **5**.

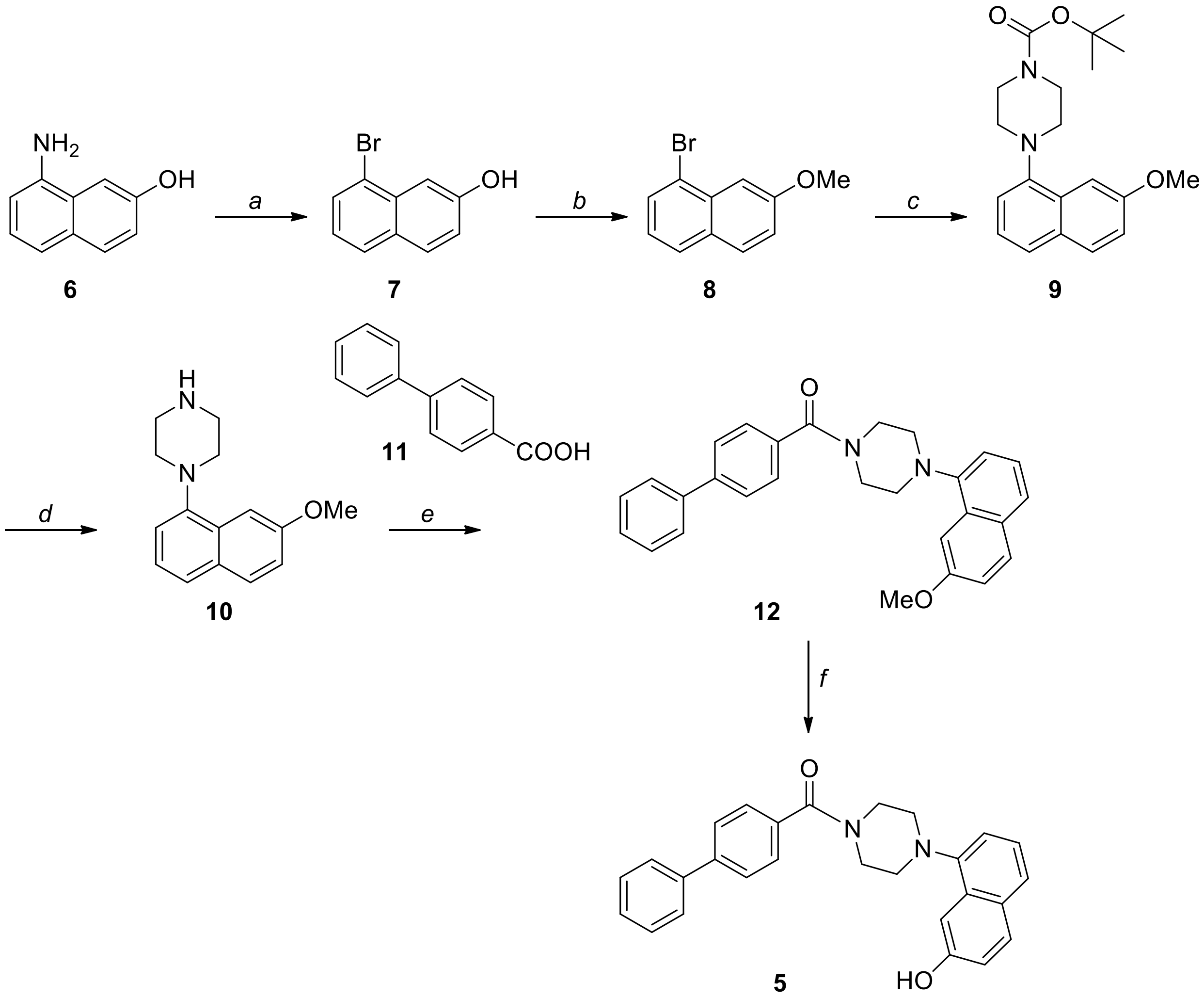

Scheme 1. *Reagents and conditions*: a) CH_3CN, HBr 48%, $NaNO_2$, CuBr, rt; b) MeI, K_2CO_3, DMF, rt; c) *N*-Boc-piperazine, Pd_2dba_3, rac-BINAP, NaO*t*Bu, toluene, 100 °C; d) TFA, DCM, 0 °C to rt; e) HATU, DIPEA, DMF, rt; f) 1 M BBr_3, CH_2Cl_2, −10 to 0 °C.

8-Bromonaphthalen-2-ol (7). To a solution of commercially available 8-amino-2-naphthol **6** (1 g, 1 equiv) in acetonitrile (7.5 mL) was added dropwise 48% hydrobromic acid (2.1 mL) and stirred at room temperature. Sodium nitrite (1.1 equiv) and CuBr (2.5 equiv) were subsequently added and then the reaction mixture was stirred overnight. Once starting material was completely reacted (TLC), the mixture was filtered through a small pad of Celite and washed with ethyl acetate. Then the filtrate was washed with brine, dried over Na_2SO_4, filtered, and evaporated in vacuo. The residue was

purified by flash column chromatography (silica gel, eluent mixture: *n*-hexane/EtOAc 9:1 to afford bromide **7** (452 mg, 32 % yield). ^{1}H-NMR ($CDCl_3$) δ (ppm): 5.20 (exchangeable s, 1H), 7.15 (dd, 1H, *J* = 8.8, 2.6 Hz), 7.18 (t, 1H, *J* = 7.7 Hz), 7.56 (d, 1H, *J* = 2.6 Hz), 7.71-7.78 (m, 3H).

*1-Bromo-7-methoxynaphthalene (**8**).* A solution of phenolic derivative **7** (425 mg, 1 equiv) in 8.5 mL of DMF was treated with anhydrous K_2CO_3 (1.5 equiv) and iodomethane (1.5 equiv) and the reaction mixture was stirred at room temperature for 24 h. The mixture was diluted with ethyl acetate and the organic extract was repeatedly washed with brine. The solvent was removed under vacuum on a rotary evaporator, affording the pure desired compound **8** (428 mg, 95 % yield), that was used in the next step without further purification. ^{1}H-NMR ($CDCl_3$) δ (ppm): 3.98 (s, 3H), 7.18 (dd, 1H, *J* = 9.0, 2.6 Hz), 7.19 (dd, 1H, *J* = 8.2, 7.5 Hz), 7.51 (d, 1H, *J* = 2.6 Hz), 7.71-7.77 (m, 3H).

*tert-Butyl 4-(7-methoxynaphthalen-1-yl)piperazine-1-carboxylate (**9**).* Commercially available 1-boc-piperazine (2 equiv), compound **8** (87.0 mg, 1 equiv) dissolved in toluene (1.3 mL), (±)-BINAP (0.10 equiv), Pd_2dba_3 (0.05 equiv) and sodium *tert*-butoxide (3 equiv) were heated in a sealed vial at 100 °C for 24 h. The reaction mixture was cooled to room temperature, diluted with EtOAc, filtered through a pad of Celite and concentrated under vacuum. Purification by silica gel column chromatography by using *n*-hexane/EtOAc 9:1 as the eluent mixture yielded **9** (110 mg, 87 % yield). ^{1}H-NMR ($CDCl_3$) δ (ppm): 1.51 (s, 9H), 2.60-3.50 (bm, 6H), 3.80-4.30 (bm, 2H), 3.94 (s, 3H), 7.07 (dd, 1H, *J* = 7.6, 0.8 Hz), 7.15 (dd, 1H, *J* = 9.0, 2.7 Hz), 7.28 (t, 1H, *J* = 7.7 Hz), 7.50-7.55 (m, 2H), 7.74 (d, 1H, *J* = 8.9 Hz).

*1-(7-Methoxynaphthalen-1-yl)piperazine (**10**).* Compound **9** (100 mg, 1 equiv) was dissolved in CH_2Cl_2 (0.91 mL), cooled to 0 °C, treated with trifluoroacetic acid (0.55 mL), and stirred at rt until consumption of starting material (TLC, 2 h). The mixture was concentrated to dryness under reduced pressure, diluted with water and the pH was adjusted to neutrality by carefully adding 1 M $NaHCO_3$ solution, then the mixture was extracted with EtOAc, and the organic phase was washed with brine. The organic layer was dried over Na_2SO_4 and concentrated to give the title compound **10** (65.4 mg, 92 % yield). ^{1}H-NMR ($CDCl_3$) δ (ppm): 2.90-3.20 (bm, 8H), 3.95 (s, 3H), 7.10 (dd, 1H, *J* = 7.4, 1.0

Hz), 7.14 (dd, 1H, J = 8.9, 2.6 Hz), 7.28 (t, 1H, J = 7.5 Hz), 7.50 (d, 1H, J = 8.1 Hz), 7.56 (d, 1H, J = 2.6 Hz), 7.74 (d, 1H, J = 8.9 Hz).

*[1,1'-Biphenyl]-4-yl(4-(7-methoxynaphthalen-1-yl)piperazin-1-yl)methanone (**12**).* HATU (1.05 equiv) was added to a solution of **11** (49.1 mg, 1 equiv) in dry DMF (1.1 mL), then DIPEA (4 equiv) was added dropwise. The resulting mixture was stirred at room temperature for 30 min, and then amine **10** (60.0 mg, 1 equiv) was added and left under stirring at room temperature until consumption of starting material (TLC). After this time, the residue was diluted with EtOAc, the organic layer was repeatedly washed with brine and dried over Na_2SO_4 and the solvent was removed under reduced pressure. The residue was purified with a flash column chromatography (silica gel, *n*-hexane/ethyl acetate 75:25), and pure fractions containing the desired compound were evaporated to dryness, affording the amide **12** (84.7 mg, 81 % yield). ^{1}H-NMR ($CDCl_3$) δ (ppm): 2.70-3.60 (bm, 8H), 3.95 (s, 3H), 7.11 (dd, 1H, J = 7.4, 0.9 Hz), 7.17 (dd, 1H, J = 9.0, 2.5 Hz), 7.29 (t, 1H, J = 7.8 Hz), 7.39 (tt, 1H, J = 7.3, 1.7 Hz), 7.44-7.50 (m, 2H), 7.53-7.59 (m, 4H), 7.59-7.63 (m, 2H), 7.67 (AA'XX', 2H, J_{AX} = 8.4 Hz, $J_{AA'/XX'}$ = 1.7 Hz), 7.76 (d, 1H, J = 8.9 Hz).

*[1,1'-Biphenyl]-4-yl(4-(7-hydroxynaphthalen-1-yl)piperazin-1-yl)methanone (**5**).* A solution of compound **12** (75.0 mg, 1 equiv) in anhydrous CH_2Cl_2 (2.1 mL) was cooled to -10 °C and treated dropwise with a 1.0 M solution of BBr_3 in CH_2Cl_2 (0.56 mL) under argon. The mixture was left under stirring at the same temperature for 5 min and then at 0 °C for 1.5 h. The mixture was then diluted with water, 1 M $NaHCO_3$ solution (pH = 7) and ethyl acetate were added. The organic phase was washed with brine, dried and concentrated. The crude product was purified by flash chromatography over silica gel. Elution with *n*-hexane/EtOAc (6:4) afforded the desired compound **5** as a white solid (64.6 mg, 89 % yield). ^{1}H-NMR (DMSO-d_6) δ (ppm): 2.80-3.20 (bm, 4H), 3.50-3.80 (bm, 4H), 7.03-7.09 (m, 2H), 7.19 (t, 1H, J = 7.7 Hz), 7.41 (tt, 1H, J = 7.4, 1.5 Hz), 7.45 (d, 1H, J = 2.3 Hz), 7.46-7.53 (m, 3H), 7.56-7.61 (m, 2H), 7.69-7.80 (m, 5H), 9.73 (exchangeable s, 1H). ^{13}C-NMR (DMSO-d_6) δ (ppm): 42.27, 47.90, 52.42 (2C), 104.86, 115.34, 118.33, 122.49, 123.50, 126.70 (2C), 126.81

(2C), 127.84 (2C), 127.90, 128.82, 129.05 (2C), 129.83, 129.94, 134.82, 139.35, 141.30, 147.21, 155.25, 168.88. HPLC analysis: retention time = 13.163 min; peak area, 98% (254 nm).

Synthesis of compound **4**.

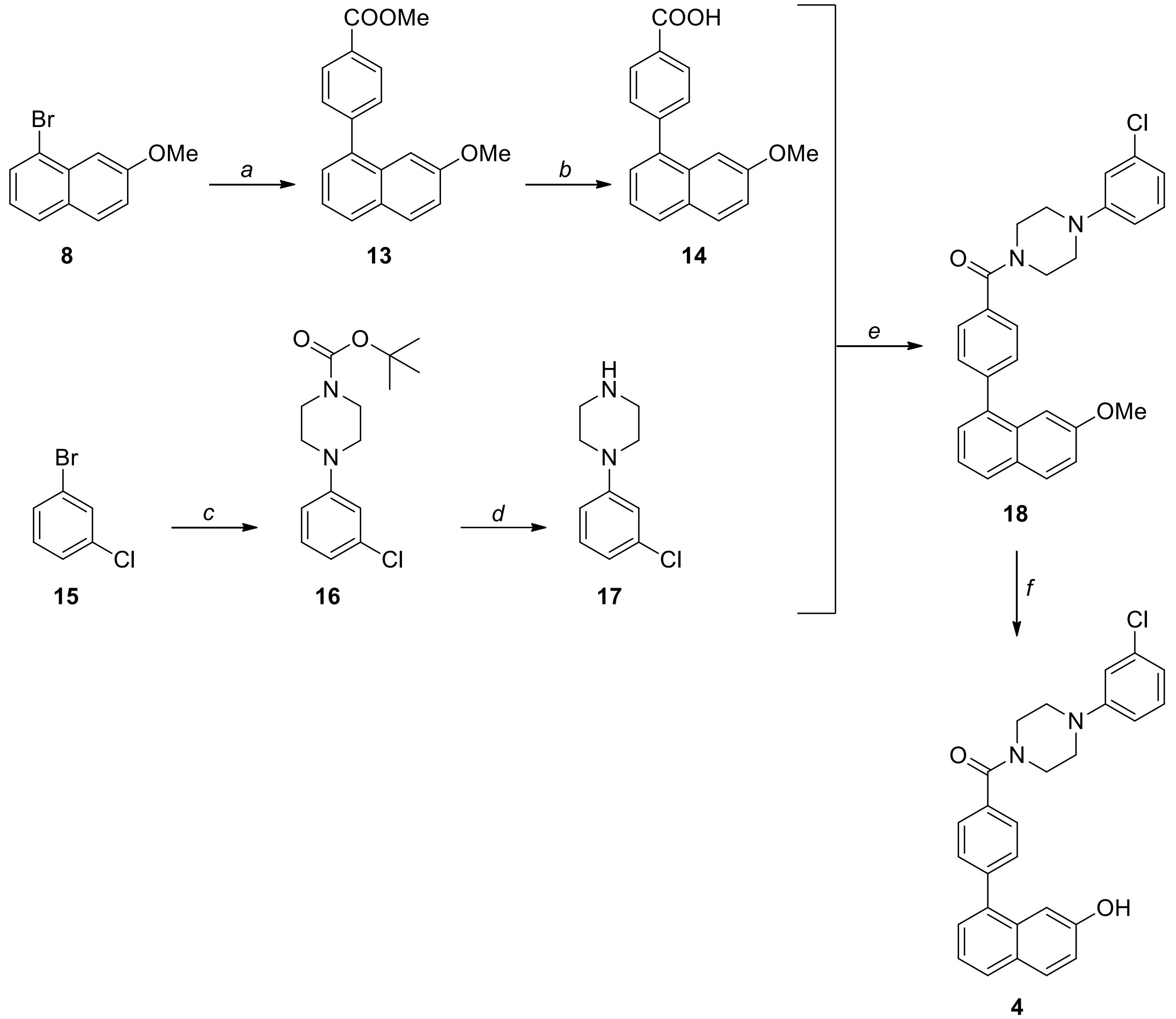

Scheme 2. *Reagents and conditions*: a) 4-COOMe-PhB(OH)$_2$, Pd(OAc)$_2$, PPh$_3$, K$_2$CO$_3$, toluene, 100 °C; b) 2 N LiOH, THF/MeOH, rt; c) *N*-Boc-piperazine, Pd$_2$dba$_3$, rac-BINAP, NaO*t*Bu, toluene, 100 °C; d) TFA, DCM, 0 °C to rt; e) HATU, DIPEA, DMF, rt; f) 1 M BBr$_3$, CH$_2$Cl$_2$, −10 to 0 °C.

*Methyl 4-(7-methoxynaphthalen-1-yl)benzoate (**13**).* A solution of $Pd(OAc)_2$ (0.03 equiv) and triphenylphosphine (0.15 equiv) in toluene (4.1 mL) was stirred at rt under argon for 10 min. After that period, compound **8** (100 mg, 1 equiv), anhydrous K_2CO_3 (1.5 equiv), and commercially available 4-(methoxycarbonyl)benzeneboronic acid (2 equiv) were sequentially added. The resulting mixture was heated at 100 °C in a sealed vial under argon for 24 h. After being cooled to rt, the mixture was diluted with water and extracted with EtOAc. The combined organic phase was washed with brine, dried and concentrated. The crude product was purified by flash chromatography over silica gel. Elution with petroleum ether/EtOAc 95:5 afforded **13** (108 mg, 88% yield). ^{1}H-NMR ($CDCl_3$) δ (ppm): 3.75 (s, 3H), 3.97 (s, 3H), 7.14 (d, 1H, J = 2.5 Hz), 7.18 (dd, 1H, J = 8.9, 2.5 Hz), 7.36-7.43 (m, 2H), 7.60 (AA'XX', 2H, J_{AX} = 8.5 Hz, $J_{AA'/XX'}$ = 1.8 Hz), 7.78-7.84 (m, 2H), 8.17 (AA'XX', 2H, J_{AX} = 8.5 Hz, $J_{AA'/XX'}$ = 1.8 Hz).

*4-(7-Methoxynaphthalen-1-yl)benzoic acid (**14**).* Methyl ester **13** (94.0 mg, 1 equiv) was dissolved in a 1:1 v/v mixture of THF/methanol (3.2 mL) and treated with 0.96 mL of 2 N aqueous solution of LiOH. The reaction was stirred overnight, then the solvents were evaporated and the residue was treated with 1 N aqueous HCl and extracted with EtOAc. The organic phase was dried and evaporated to afford the pure desired carboxylic acid derivative **14** (80.0 mg, 87 % yield). ^{1}H-NMR (acetone-d_6) δ (ppm): 3.77 (s, 3H), 7.18-7.26 (m, 2H), 7.41-7.48 (m, 2H), 7.69 (AA'XX', 2H, J_{AX} = 8.3 Hz, $J_{AA'/XX'}$ = 1.8 Hz), 7.87-7.95 (m, 2H), 8.21 (AA'XX', 2H, J_{AX} = 8.3 Hz, $J_{AA'/XX'}$ = 1.7 Hz).

*tert-Butyl 4-(3-chlorophenyl)piperazine-1-carboxylate (**16**).* Commercially available 1-boc-piperazine (2 equiv), 1-bromo-3-chlorobenzene **15** (200 mg, 1 equiv) dissolved in toluene (3.8 mL), (±)-BINAP (0.10 equiv), Pd_2dba_3 (0.05 equiv) and sodium *tert*-butoxide (3 equiv) were heated in a sealed vial at 100 °C for 24 h. The reaction mixture was cooled to room temperature, diluted with EtOAc, filtered through a pad of Celite and concentrated under vacuum. Purification by silica gel column chromatography by using petroleum ether/EtOAc 95:5 as the eluent mixture yielded **16** (306 mg, 99 % yield). ^{1}H-NMR ($CDCl_3$) δ (ppm): 1.48 (s, 9H), 3.15 (t, 4H, J = 5.1 Hz), 3.59 (t, 4H, J = 5.2 Hz), 6.79-6.93 (m, 3H), 7.18 (t, 1H, J = 8.1 Hz).

*1-(3-Chlorophenyl)piperazine (**17**).* Compound **16** (520 mg, 1 equiv) was dissolved in CH_2Cl_2 (5.5 mL), cooled to 0 °C, treated with trifluoroacetic acid (3.3 mL), and stirred at rt until consumption of starting material (TLC, 2 h). The mixture was concentrated to dryness under reduced pressure, diluted with water and the pH was adjusted to neutrality by carefully adding 1 M $NaHCO_3$ solution, then the mixture was extracted with EtOAc and the organic phase was washed with brine. The organic layer was dried over Na_2SO_4 and concentrated to give the title compound **17** (315 mg, 91 % yield). ^{1}H-NMR ($CDCl_3$) δ (ppm): 2.98-3.17 (m, 5H), 3.22-3.27 (m, 4H), 6.79 (ddd, 1H, J = 8.4, 2.4, 0.8 Hz), 6.85 (ddd, 1H, J = 7.8, 1.9, 0.8 Hz), 6.88 (t, 1H, J = 2.2 Hz), 7.18 (t, 1H, J = 8.1 Hz).

*(4-(3-Chlorophenyl)piperazin-1-yl)(4-(7-methoxynaphthalen-1-yl)phenyl)methanone (**18**).* HATU (1.05 equiv) was added to a solution of **14** (75.0 mg, 1 equiv) in dry DMF (1.2 mL), then DIPEA (4 equiv) was added dropwise. The resulting mixture was stirred at room temperature for 30 min and then amine **17** (53.0 mg, 1 equiv) was added and left under stirring at room temperature until consumption of starting material (TLC). After this time, the residue was diluted with EtOAc, the organic layer was repeatedly washed with brine and dried over Na_2SO_4 and the solvent was removed under reduced pressure. The residue was purified with a flash column chromatography (silica gel, *n*-hexane/ethyl acetate 7:3), and pure fractions containing the desired compound were evaporated to dryness, affording the amide **18** (102 mg, 83 % yield). ^{1}H-NMR ($CDCl_3$) δ (ppm): 3.15-3.40 (m, 4H), 3.70-4.08 (bm, 4H), 3.78 (s, 3H), 6.87 (dd, 1H, J = 8.5, 2.2 Hz), 6.88-6.93 (m, 1H), 6.95 (t, 1H, J = 2.0 Hz), 7.16-7.24 (m, 3H), 7.35-7.43 (m, 2H), 7.95 (s, 4H), 7.79-7.85 (m, 2H).

*(4-(3-Chlorophenyl)piperazin-1-yl)(4-(7-hydroxynaphthalen-1-yl)phenyl)methanone (**4**).* A solution of compound **18** (91.0 mg, 1 equiv) in anhydrous CH_2Cl_2 (2.3 mL) was cooled to -10 °C and treated dropwise with a 1.0 M solution of BBr_3 in CH_2Cl_2 (0.63 mL) under argon. The mixture was left under stirring at the same temperature for 5 min and then at 0 °C for 2 h. The mixture was then diluted with water, 1 M $NaHCO_3$ solution (pH = 7) and ethyl acetate were added. The organic phase was washed with brine, dried and concentrated. The crude product was purified by flash chromatography over silica gel. Elution with *n*-hexane/EtOAc (6:4) afforded the desired compound **4** as a white solid (68.7

mg, 78 % yield). ^{1}H-NMR (DMSO-d_6) δ (ppm): 3.31 (s, 4H), 3.50-3.90 (bm, 4H), 6.83 (dd, 1H, J = 7.8, 1.2 Hz), 6.94 (dd, 1H, J = 8.3, 1.9 Hz), 7.00 (t, 1H, J = 2.1 Hz), 7.08-7.13 (m, 2H), 7.24 (t, 1H, J = 8.1 Hz), 7.33-7.38 (m, 2H), 7.52-7.57 (m, 2H), 7.57-7.62 (m, 2H), 7.81-7.88 (m, 2H), 9.72 (exchangeable bs, 1H). ^{13}C-NMR (DMSO-d_6) δ (ppm): 47.89 (4C), 106.40, 114.08, 115.03, 118.52, 118.56, 122.25, 127.05, 127.23 (2C), 127.74, 128.07, 129.63 (2C), 129.99, 130.47, 132.33, 133.84, 134.51, 136.84, 141.83, 151.94, 155.80, 168.90. HPLC analysis: retention time = 13.622 min; peak area, 97% (254 nm).

Table S1. MM-PBSA results for the complexes of MAGL with the two hypothesized binding orientations (binding mode A and B) of compound **1**. ΔPBSA is the sum of the electrostatic (ELE), van der Waals (VDW), polar (EPB) and non-polar (ENPOLAR) solvation free energy. Data are expressed as kcal•mol^{-1}.

	**ELE**	**VDW**	**ENPOLAR**	**EPB**	**ΔPBSA**
**A**	-13.9	-52.1	-4.8	40.7	-30.1
**B**	-17.8	-49.9	-5.0	44.1	-28.6

References.

(1) Granchi, C.; Rizzolio, F.; Palazzolo, S.; Carmignani, S.; Macchia, M.; Saccomanni, G.; Manera, C.; Martinelli, A.; Minutolo, F.; Tuccinardi, T. Structural Optimization of 4-Chlorobenzoylpiperidine Derivatives for the Development of Potent, Reversible, and Selective Monoacylglycerol Lipase (MAGL) Inhibitors. *J. Med. Chem.* **2016**, *59* (22), 10299–10314.
